# UCP2 silencing restrains leukemia cell proliferation through glutamine metabolic remodeling

**DOI:** 10.3389/fimmu.2022.960226

**Published:** 2022-10-06

**Authors:** Tiphaine Sancerni, Ophélie Renoult, Angèle Luby, Cédric Caradeuc, Véronique Lenoir, Mikael Croyal, Céline Ransy, Esther Aguilar, Catherine Postic, Gildas Bertho, Renaud Dentin, Carina Prip-Buus, Claire Pecqueur, Marie-Clotilde Alves-Guerra

**Affiliations:** ^1^ Université Paris Cité, CNRS, INSERM, Institut Cochin, Paris, France; ^2^ Nantes Université, INSERM, CNRS, CRCI2NA, Nantes, France; ^3^ Université Paris Cité, CNRS UMR8601, Paris, France; ^4^ Asociación Española Contra el Cáncer (AECC), Fundación Científica AECC, Madrid, Spain

**Keywords:** metabolism rewiring, leukemia, mitochondria, glutamine, UCP2, metabolite carrier

## Abstract

T-cell acute lymphoblastic leukemia (T-ALL) is an aggressive hematologic malignancy derived from early T cell progenitors. Since relapsed T-ALL is associated with a poor prognosis improving initial treatment of patients is essential to avoid resistant selection of T-ALL. During initiation, development, metastasis and even in response to chemotherapy, tumor cells face strong metabolic challenges. In this study, we identify mitochondrial UnCoupling Protein 2 (UCP2) as a tricarboxylic acid (TCA) cycle metabolite transporter controlling glutamine metabolism associated with T-ALL cell proliferation. In T-ALL cell lines, we show that UCP2 expression is controlled by glutamine metabolism and is essential for their proliferation. Our data show that T-ALL cell lines differ in their substrate dependency and their energetic metabolism (glycolysis and oxidative). Thus, while UCP2 silencing decreases cell proliferation in all leukemia cells, it also alters mitochondrial respiration of T-ALL cells relying on glutamine-dependent oxidative metabolism by rewiring their cellular metabolism to glycolysis. In this context, the function of UCP2 in the metabolite export of malate enables appropriate TCA cycle to provide building blocks such as lipids for cell growth and mitochondrial respiration. Therefore, interfering with UCP2 function can be considered as an interesting strategy to decrease metabolic efficiency and proliferation rate of leukemia cells.

## Introduction

T acute lymphoblastic leukemia (T-ALL) is an aggressive hematologic tumor characterized by a high rate of lymphoblast proliferation. Oncogenic NOTCH signaling resulting from activating mutations of NOTCH1 is a major driver of T-ALL transformation. An important issue of T-ALL treatment is tumor relapse due to the selection and adaption of a clonal population resistant to treatment (1). Relapsed T-ALL is associated with a poor prognosis and therefore, improvement of the initial treatment of patients is essential to avoid selection of resistant T-ALL.

Increased proliferation and metabolism reprogramming are key features of cancer cells (2, 3). In most cancer cells derived from epithelium, the metabolic shift from oxidative metabolism to glycolysis (Warburg effect) sustains cell proliferation, ensuring sufficient precursors for cell biogenesis (4, 5). However, although tumors have long been considered only glycolytic, recent studies have shown that some tumors may also adopt oxidative metabolism (6–8). Indeed, tumor cells require large amounts of energy and carbon skeletons for building blocks in order to maintain a high rate of proliferation. Therefore, they are dependent on both the cellular oxidative capacities and the regeneration of intermediate metabolites (9). In addition, cell metabolism can influence resistance to chemotherapy as well as the aggressiveness of cancer (10). Modulating metabolic reprogramming therefore appears to be a potential therapeutic strategy in cancer.

UnCoupling Protein 2 (UCP2) belongs to a large family of mitochondrial transporters of the mitochondrial inner membrane (11). The generation of UCP2 knock-out mice provided information on the role of UCP2 *in vivo*. It has been shown that the lack of UCP2 increases immunity with a protective role against pathogens thanks to an increase in the oxidative burst of macrophages (12, 13). It is important to note that a negative impact of the loss of UCP2 has also been demonstrated with regard to autoimmune diseases [atherosclerosis (14), type 1 diabetes (15) and multiple sclerosis (16)]. UCP2 also increased the rate of cell division in primary mouse fibroblasts and CD3-activated T cells and altered the balance between respiration and glycolysis as energy providers (17). Overall, the role of UCP2 on metabolism, oxidative stress and cell division rate defines this protein as a relevant target for cancer treatments. Consistently, the overexpression of UCP2 in melanoma cells reduced their rate of proliferation and induced metabolic remodeling towards oxidation (18, 19). Likewise, loss of UCP2 rendered colon cells more prone to malignant transformation through metabolic reprogramming and disruption of redox homeostasis, promoting worse outcomes in colorectal cancer (20). However, studies in other models of epithelial cancer, such as the skin (21), breast (22) and lung (23), show an opposite relationship between UCP2 and cancer proliferation. The introduction of UCP2 in liposomes allowed to evaluate its transport activity and to propose that UCP2 can export four-carbon metabolites such as fumarate, malate, oxaloacetate and aspartate from the mitochondrial matrix, facilitating the TCA cycle and regulating metabolism and ROS production (24, 25). Thus, UCP2 may exert a dual role and limit or accelerate tumor initiation and progression depending on bioenergetics resources, initial needs and metabolic fluxes. In T-ALL leukemia cells, activation of Notch 1 signaling pathway renders these cells highly dependent on glutamine (26). Strikingly, glutamine counteracts the translational repression of *Ucp2* (27) and UCP2 plays a role in glutamine oxidation in macrophages (28). Due to this dual dependency, UCP2 appears as an attractive candidate in the metabolic remodeling of T-ALL leukemia cells.

The aim of our study was to decipher how UCP2 acts in the control of metabolism associated with proliferation in T-ALL cells. Our data show that T-ALL cell lines differ by their substrate dependency and their energetic metabolism (glycolysis and oxidative). We took advantage of these different T-ALL cell lines to study the role of UCP2 in glutamine metabolism and proliferation. In T-ALL cell lines, we show that UCP2 expression is controlled by glutamine metabolism and is essential for their proliferation. Our data demonstrate that in T-ALL cells, the function of UCP2 in the metabolite export of malate enables optimal function of the glutamine-dependent TCA cycle, ensuring oxidation of energy substrates and providing building blocks such lipids for proliferation. Interestingly, our results show that glutaminergic HPB-ALL cells reprogram their metabolism upon metabolic challenges in a UCP2-dependent manner, in contrast to glycolytic Jurkat cells, which adapt their metabolism independently of UCP2 expression. These results highlight a specific function of UCP2 upon metabolic stress based on the intrinsic metabolic plasticity of the T-ALL cells. Therefore, interference with UCP2 function can be considered as a strategy to decrease the metabolic efficiency and the proliferation rate of leukemic T-ALL cells.

## Material and methods

### Cell culture, treatments and doubling time

Jurkat and HPB-ALL malignant human T-ALL Cells (courtesy of Pfumio’s laboratory) were cultured in RPMI-1640 GlutaMAX™ medium (ThermoFisher scientific^®^), supplemented with 10% (v/v) decomplemented fetal bovine serum (FBS) and 1% (v/v) Pen/Strep at 37°C and 5% carbon dioxide. Cells were maintained in the growth phase.

The cells were seeded in RPMI-1640 GlutaMAX™ medium or RPMI-1640 no glutamine medium (with addition of dimethyl-αKetoglutarate (DM-αKG or KG) (2 mM, Sigma-Aldrich), dimethyl-Malate (DM-Mal or MAL) (5 mM, Sigma-Aldrich) or RPMI-GlutaMAX™ (with addition of BPTES (10 µM, SML0601 Sigma-Aldrich), 6-Diazo-5-oxo-L-norleucine (DON) (0.05 µg/mL, D2141 Sigma-Aldrich), L-Asparaginase (L-ASNase) (0.05 U/mL (Jurkat cells) and 0.01 U/mL (HPB-ALL cells) A3809 Sigma-Aldrich) at optimal concentrations: 0.6*10^6^ cells/mL for Jurkat cells and 1.0 million cells/mL for HPB-ALL cells. They were counted at 24 hours, 48 hours and 72 hours on 0.4% (v/v) Trypan blue (Sigma-AldrichT8154) with Kova Glasstic Slides. They were collected after 72 hours for oroboros experiments and western blots analyzes.

For the labeling experiments, 10 million cells were grown in six wells in glutamine-free RPMI with 2 mM [U-^13^C] glutamine, or in glucose- and glutamine-free RPMI with 11 mM [U-^13^C] glucose or with 3 mM [U-^13^C] malate (Cambridge Isotope Laboratories) for 6 hours to reach isotopic steady state. The cells were harvested and frozen at -80°C.

### Western blot analysis

After the treatments indicated, the cells were harvested and the proteins extracted with lysis buffer (1.5 mmol/L EDTA, 50 mmol/L Hepes pH 7.4, 150 mmol/L NaCl, 10% (v/v) glycerol, and 1% (v/v) NP40) containing proteases and phosphatase inhibitors (Thermofisher scientific). The lysates were centrifuged at 12, 000 g for 20 minutes at 4°C and the supernatants were collected. Equal amounts of proteins (40 µg) were separated onto a 4% to 20% SDS-PAGE gel (Bio-Rad), transferred onto a nitrocellulose membrane (Protran, Whatman) at 70 V, 35 minutes at 4°C and blocked with 5% w/v skimmed milk or BSA for 1 hour. The antibodies used in immunoblotting were as follows: homemade anti-UCP2 (UCP2–605) (29), anti-Notch1^ICD^ valine1744 (Cell signaling, 4147); anti-cMyc (Cell Signaling, 5605); anti-HK2 (Cell signaling, 2867); anti-PDH (Cell signaling, 2784); anti-GLS (Abcam, ab93434); anti-GS (BD, 610517); anti-CS (Abcam, ab85669); anti-GADPH (Cell Signaling, 2118); anti-ASNS (proteintech, 14681-1-AP) and anti-ß-Actin (Sigma-Aldrich, A5441), anti-α-Tubulin (Sigma-Aldrich, T5168). A direct recording of the chemiluminescence and a quantification (Fusion^®^ software) were carried out. Western blot analyzes were performed using independent samples.

### Mitochondrial respiration measurements

#### High-resolution respirometry with Oroboros device

The oxygen consumption rate (OCR) by the T-ALL cells was measured on fresh medium by a high‐resolution 2 mL glass chamber respirometer (Oroboros Oxygraph‐2k). The electrode was calibrated at 37°C, 100% and 0% oxygen before adding 2 mL of cells (5.5 * 10^6^ cells /mL) to each chamber and the flux of O_2_ consumption was measured in pmol/s. Oligomycin (0.5 μg/mL) was added to block complex V, as an estimate of the contribution of mitochondrial leakage to overall cellular respiration. Increasing amounts of carbonyl cyanide m-chlorophenyl hydrazone (CCCP) were added to measure maximum respiratory capacity. Finally, potassium cyanide (KCN) was added to measure the non-mitochondrial respiration.

#### Respiration measurement with SeaHorse XF96 equipment

The T-ALL cells were cultured in RPMI 1640 (GIBCO # 11875-085) supplemented with 10% v/v decomplemented FBS at 37°C, 5% CO_2_. XFp 96-well plates were coated with 20 μL of 100 μg/mL poly D lysine (PDL) solution (in H_2_O) for 1 hour, then washed twice with 200 μL of sterile water. They were dried in a culture hood for 1 hour. Then, 80 μL of RPMI XF pH 7.4 assay medium (added with 2 mg/mL final glucose and 2 mM glutamine if necessary) were added per well and the plate (without cells) was pre-incubated at 37°C for 1 hour. Then, 3.0*10^5^ T-ALL cells (40 μL) were seeded per well coated with PDL. The plates were centrifuged for 1 minute at 200 g to adhere the cells to the plate and 100 μL of assay medium was added to each well.

Mitochondrial respiration was measured after addition of DM-αKG or DM-Mal, or the corresponding medium (for control); then the uncoupling respiration was measured after addition of oligomycin (1 µg/mL), the maximum respiration was measured after addition of CCCP (3 µM final for HPB-ALL cells and 0.5 µM final for Jurkat cells) and finally antimycin + rotenone (1 µg/mL) was added to determine non-mitochondrial respiration. The oxygen consumption rate (OCR) and extracellular acidification rate (ECAR) were measured all along the run. The OCR/ECAR ratio indicates the cellular preference for OXPHOS over glycolysis.

### UCP2 CRISPR/Cas9 cells generation

pLentiCRISPR v2 13kb containing gRNA with the following sequence: CTACAAGACCATTGCCCGAG, was purchased from GenScript USA Inc (NJ). The pLentiCRISPR v2 vector expresses both Cas9-nickase protein and puromycin resistance for selection. Gisèle Froment, Didier Nègre and Caroline Costa produced lentiviruses from the lentivector production facility of SFR Biosciences (UMS3444/CNRS, US8/Inserm, ENS de Lyon, UCBL). In 200 µL of RPMI GlutaMAX™, 5*10^5^ HPB-ALL or Jurkat cells were infected with the UCP2^CRISPR^ lentivirus for 3 hours at 37°C, 5% CO_2_. The cells remained in culture for 4 days then selected with puromycin (1 µM for HPB-ALL cells and 0.5 µM for Jurkat cells) for 8 days. At least, two selected CRISPR populations from different infections were analyzed.

### LC-HRMS

The dried samples were deproteinized by adding methanol and centrifuged 10 minutes at 10,000 g. The supernatants were divided to investigate both organic acids and amino acids enrichment. For organic acids, supernatants were dried using nitrogen vapor before being recovered in water containing 0.1% v/v formic acid. Amino acid fractions were dried and esterified with butanol 5% v/v chloride acetyl and incubated 30 minutes at 60°C. Samples were dried and solubilized in water 0.1% v/v formic acid. All samples were injected into the Liquid Chromatography-High Resolution Mass Spectrometry (LC-HRMS) system by a Synapt G2 HMRS Q-TOF mass spectrometer with an electrospray interface operating in negative (organic acid) and positive mode (amino acid) and an Acquity H-Class^®^ UPLCTM device (Waters Corporation). Data acquisition and integration were performed with MassLynx^®^ and TargetLynx^®^1 version 4.1 software, respectively (Waters Corporation). The data enrichment analyzes were carried out with Isocor Software version 2.0 (30).

### NMR

Ten HPB-ALL parental cell culture media and 10 HPB-ALL UCP2^CRISPR^ cell culture media (each from 24 hours culture) were analyzed. Furthermore, 10 HPB-ALL and UCP2^CRISPR^ HPB-ALL cell pellets were analyzed after specific extraction of the aqueous phase. The 1H-Nuclear Magnetic Resonance (NMR) spectra were measured at 300K on a Bruker Avance II 500MHz spectrometer (Bruker BioSpin, France) equipped with a SampleXpress automation sample changer and a 5 mm cryogenic DCH probe with Z-gradient. For analysis of the exometabolome (extracellular environment), each 5 mm NMR tube was filled with 432 µL of culture medium and 168 µL of a 100 mM phosphate buffer pH 7.4 in heavy water (D_2_O) containing 1mM TSP as a 1H-NMR reference of chemical shift at -0.016 ppm. For the analysis of the endometabolome (intracellular compartment), cell pellets containing 10*10^6^ cells were prepared for the extraction of the metabolites. Cell pellets were treated with 400 µL of ice-cold methanol and 65 µL of ice water. A volume of 200 µL of ice-cold chloroform, then 200 µL of ice-cold water and finally 200 µL of ice-cold chloroform were further added. The samples were then left on ice for 15 minutes, and centrifugated at 2,000 g for 15 minutes. The upper methanol/water phase (containing polar metabolites) was transferred to glass vials and placed under a stream of nitrogen for 30 minutes. These vials were freeze-dried overnight. The dry samples were resuspended in 580 µL of NMR buffer (100 mM sodium phosphate buffer pH 7.4, in D_2_O, with 3.57 mM TSP and 6 mM NaN3) to obtain the intracellular polar phase extract. The spectra acquisition was based on already published protocols for biofluids and tissue extracts, respectively (31). The data processing was performed using TopSpin 3.1 (Bruker BioSpin, France). Additional processing and bucketing of the 1H-NMR spectra was performed using NMRProcFlow (32). An overview of the results was generated with unsupervised analysis using the PCA method to identify outliers and perform quality control on the data. Based on these results, group classifications were established from the 20 samples (10 parental HPB-ALL cells and 10 UCP2^CRISPR^ HPB-ALL cells) to generate a supervised analysis. The O-PLS-DA models were calculated to identify the discriminating metabolites from the VIP plot. Univariate data analysis was performed using MetaboAnalyst (33) to calculate p-values and generate boxplots of relevant variables.

### Statistical analysis

The non-parametric Mann-Whitney U-test and ANOVA using GraphPad Prism 5 and 9 software analyzed the differences between the experimental groups. The correlations were studied by the Spearman test. The results are expressed as the mean ± SEM.

## Results

### T-ALL cell lines exhibiting different UCP2 steady-state expression differ in doubling time and oxidative capacities

**Figure 1:**
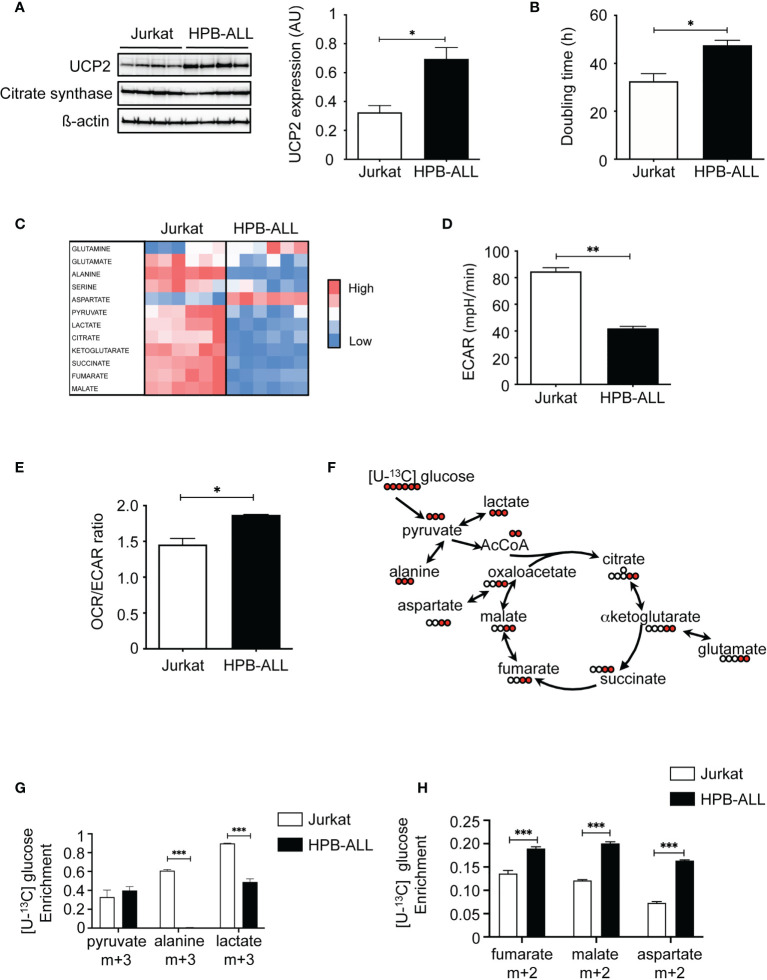
T-ALL cell lines exhibiting different UCP2 steady-state expression differ in doubling time and oxidative capacities. **(A)** UCP2, citrate synthase (CS) and ß-actin immunoblots in whole cell extracts of Jurkat, and HPB-ALL cell lines cultured in complete medium (n = 4). Quantification of UCP2 steady-state expression normalized to ß-actin; data are expressed as mean ± SEM (n = 4). **(B)** Doubling time in hours evaluated for 72 hours of culture in complete medium; data represent mean ± SEM (n = 4). **(C)** The relative abundance of 12 metabolites measured by liquid chromatography–tandem mass spectrometry (LC-HRMS) is visualized as a heat map representation (blue color corresponds to low values and red to high values) by distinguishing the number of samples. Jurkat cells and HPB-ALL cells were cultured in complete medium. **(D)** Measurement of ECAR (mpH/min) from the Jurkat and HPB-ALL cell lines. Data are expressed as mean ± SEM (n = 6). **(E)** Basal energy metabolism in complete medium was assessed by analyzing the OCR/ECAR ratio measurement with SeaHorse XF96 equipment in Jurkat and HPB-ALL cells; data are expressed as mean ± SEM (n = 3). **(F)** Representation of the labeling scheme using [U-^13^C] glucose. For simplicity, the labeling of the first round in the TCA cycle is displayed (red). **(G)** Enrichment in (m+3) pyruvate, (m+3) alanine and (m+3) lactate indicates the part derived from the oxidation of [U-^13^C] glucose in Jurkat and HPB-ALL cells; data are expressed as mean ± SEM (n = 3). **(H)** The enrichment in (m+2) fumarate, (m+2) malate and (m+2) aspartate indicates the part derived from the oxidation of [U-^13^C] glucose in Jurkat and HPB-ALL cells; data are expressed as mean ± SEM (n = 3). Statistical significance is represented as follows: ***p<0.001, **p<0.01, *p<0.05.

We first characterized T-ALL cell lines for UCP2 expression and metabolism. Jurkat, HPB-ALL, DND41 and MOLT4 are various T-ALL cell lines sharing altered Notch signaling as their common oncogenic trigger. Interestingly, they differed for the steady-state protein expression of UCP2 (**Figure 1A** and **Supplementary Figure 1A**). In particular, the HPB-ALL cells expressed twice more UCP2 as compared to the Jurkat, and the other cell lines tested (**Figure 1A** and **Supplementary Figure 1A**). Differences in UCP2 expression do not result from different mitochondrial content, as these cell lines expressed similar expression of citrate synthase (CS) (**Figure 1A** and **Supplementary Figure 1A**). Jurkat cells displayed a lower doubling time compared to HPB-ALL cells (**Figure 1B**), but no significant correlation was observed between UCP2 levels and doubling time when including all cell lines (**Supplementary Figure 1B**). Interestingly, we found a positive correlation between UCP2 levels and maximal respiration (**Supplementary Figure 1C**), which highlights the dependence between oxidative capacity and UCP2 levels. We then focused our experiments on Jurkat and HPB-ALL cells because the two cell lines differed significantly in their UCP2 steady state (**Figure 1A**) and interestingly, Jurkat cells displayed a lower doubling time to that of HPB-ALL cells (**Figure 1B**). These two T-ALL cell lines expressing different levels of UCP2 provided a means to address the influence of UCP2 on two important aspects of T-ALL cell biology: proliferation rate and oxidative capacity. We then performed metabolic analyses of twelve metabolites using Liquid Chromatography-High Resolution Mass Spectrometry (LC-HRMS). These metabolites extracted from each specimen cultured in complete media revealed that overall Jurkat cells displayed a distinct global metabolism as compared to HPB-ALL cell line (**Figure 1C**), which prompted us to deeper investigate these metabolic differences. An increase in the rate of extracellular acidification (ECAR) in Jurkat cells compared to HPB-ALL was observed, indicating that Jurkat cells were more actively using glycolysis to produce lactate (**Figure 1D**). In contrast, an increase in the OCR/ECAR ratio was observed for the HPB-ALL cells, indicating that they mainly rely on oxidative metabolism (**Figure 1E** and **Supplementary Figure 2C**). To better characterize the respective proportion of glucose used to fuel glycolysis or oxidative TCA cycle, we performed [U-^13^C] glucose fluxomics experiments and analyzed isotopic enrichment of (m+3) isotopologues of pyruvate, alanine and lactate, as well as (m+2) isotopologues of TCA metabolites (**Figures 1F, G**). In Jurkat cells, more than 60% of alanine and 90% of lactate directly derived from glucose. In contrast, in HPB-ALL cells, (m+3) alanine was undetectable and only 50% of (m+3) lactate was measured in HPB-ALL cells (**Figure 1G**). In agreement with these results, higher HK2 protein expression and higher intracellular amounts of pyruvate, alanine and lactate were observed in Jurkat cells as compared to HPB-ALL cells (**Supplementary Figures 2A, B**). In contrast, and in agreement with their increased mitochondrial respiration (**Supplementary Figure 2C**), HPB-ALL cells displayed higher enrichment of (m+2) isotopologues of fumarate, malate and aspartate from [U-^13^C] glucose compared to Jurkat cells (**Figure 1H**). Fluxomics experiments using [U-^13^C] glutamine also revealed glutamine metabolic discrepancies between the two cell lines. First, anaplerotic fueling of the TCA cycle by glutamine was increased in HPB-ALL cells as shown by higher enrichment of (m+4) citrate and higher enrichment of (m+2) isotopologues of fumarate, malate and aspartate (**Supplementary Figures 2C-F**). Interestingly, the mitochondrial fate of glutamine-derived αketoglutarate also differed between the two cell lines, as shown by the respective enrichment of (m+4) and (m+5) citrate from [U-^13^C] glutamine measuring respectively classic TCA fueling or the reductive carboxylation through isocitrate dehydrogenase 2 (IDH2). Interestingly, in Jurkat cells, the enrichment of (m+4) citrate was roughly equal to (m+5) citrate (27% vs. 20% respectively) while in HPB-ALL cells the enrichment of (m+4) citrate was more than 3-fold higher than (m+5) citrate (35% vs. 8% respectively) (**Supplementary Figure 2D**). These results demonstrate that Jurkat cells used glutamine to perform both oxidative and reductive mitochondrial metabolism, unlike HPB-ALL cells, which primarily use glutamine to fuel the oxidative TCA cycle.

Overall, our results reveal that Jurkat cells express low levels of UCP2 and are highly glycolytic while HPB-ALL cells strongly express UCP2 and rely mainly in oxidative metabolism.

### Glutamine deprivation impacts UCP2 content, proliferation rate and metabolism differentially depending on T-ALL cells

**Figure 2:**
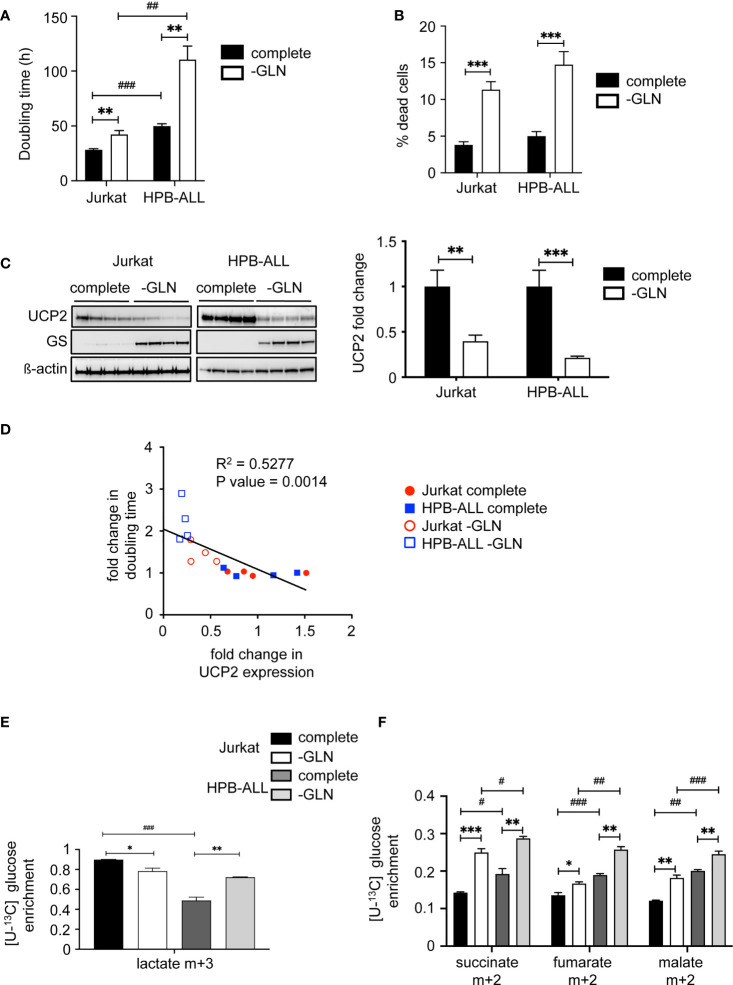
Glutamine deprivation impacts UCP2 content, proliferation rate and metabolism differentially depending on T-ALL cells. **(A)** Doubling time in hours evaluated for 72 hours of culture in complete (complete) or glutamine-deprived medium (-GLN); data represent the mean ± SEM (n = 4). **(B)** Percentage of dead cells measured by trypan blue staining. Data are expressed as mean ± SEM (n = 16). **(C)** Immunoblotting of UCP2 and glutamine synthase (GS) in whole cell extracts of Jurkat and HPB-ALL cell lines after 72 hours of incubation in complete or glutamine-deprived medium (-GLN) (n = 4); quantification of the steady state of UCP2 normalized to ß-actin and expressed as fold change between each individual value and the mean value of respective controls (cells in complete medium); data are expressed as mean ± SEM (n = 4). **(D)** Correlation between UCP2 expression and doubling time using Relative Units obtained by the ratio between each individual value and the mean value of respective controls (same cell line in complete medium) and analyzed with Spearman’s correlation test. **(E)** Enrichment of (m+3) lactate indicates the portion that derived from [U-^13^C] glucose in Jurkat and HPB-ALL cells cultured in complete medium (complete, black and dark grey respectively) (n = 6) and in glutamine deprivation medium (-GLN, white and light grey respectively) (n = 3). **(F)** Enrichment of (m+2) succinate, (m+2) fumarate and (m+2) malate indicates the portion that derived from oxidation of [U-^13^C] glucose in Jurkat and HPB-ALL cells cultured in complete medium (complete, black and dark grey respectively) (n = 6) and in glutamine deprivation medium (-GLN, white and light grey respectively) (n = 3); data are expressed as mean ± SEM. Statistical significance is represented as follows: ***p<0.001; **p<0.01; *p<0.05, and # = Jurkat vs. HPB-ALL. ^#^p<0.05 , ^##^p<0.01 and ^###^p<0.001.

Since we have previously reported in various primary and immortalized cells that (1) UCP2 expression is involved in the modulation of cell proliferation through metabolic rewiring (17, 18), and (2) the glutamine pool directly controlled UCP2 translation (27, 28), we investigated how UCP2 might be involved in the glutamine sensing response in T-ALL cells through metabolic challenge. In both cell lines, glutamine deprivation significantly decreased cell proliferation, as shown by the increase in doubling time (**Figure 2A**), and triggered cell death as measured by trypan blue staining (**Figure 2B**). As previously reported, glutamine deprivation induced the expression of glutamine synthase (34), and also significantly decreased UCP2 expression in both cell lines (**Figure 2C**). Interestingly, we observed a significant inverse correlation between relative changes in UCP2 expression *versus* doubling time (**Figure 2D**). To investigate how apparent basal metabolic discrepancies affected the metabolic response to glutamine deprivation, we performed in-depth metabolic analyzes using LC-HRMS. In Jurkat cells, glutamine deprivation resulted in a significant decrease in intracellular glutamate levels (**Supplementary Figure 3A**), as well as in all mitochondrial metabolites tested, namely succinate, fumarate, and malate (**Supplementary Figure 3B**). In these cells, no significant difference was observed in the level of intracellular lactate (**Supplementary Figure 3C**). In contrast, in HPB-ALL cells, while the level of intracellular glutamine was also markedly decreased by glutamine deprivation (**Supplementary Figure 3D**), its impact on glutamate, succinate, fumarate and malate levels was reduced compared to Jurkat cells (**Supplementary Figures 3A and 3B**). In fact, HPBALL cells reprogrammed their metabolism upon glutamine deprivation by increasing glycolysis. Indeed, [U-^13^C] glucose fluxomics experiments showed an increased conversion of [U-^13^C] glucose to both (m+3) lactate (**Figure 2E**) and (m+2) TCA intermediates (**Figure 2F**).

Altogether, our results show that glutamine deprivation reduced UCP2 expression and the proliferation rate of both cell lines. They both triggered a metabolic rewiring by redirecting pyruvate to TCA. However, glutamine deprivation further reprograms HPB-ALL metabolism towards increased glycolysis.

### Inhibition of glutamine metabolism in T-ALL cells decreases UCP2 steady-state expression

**Figure 3:**
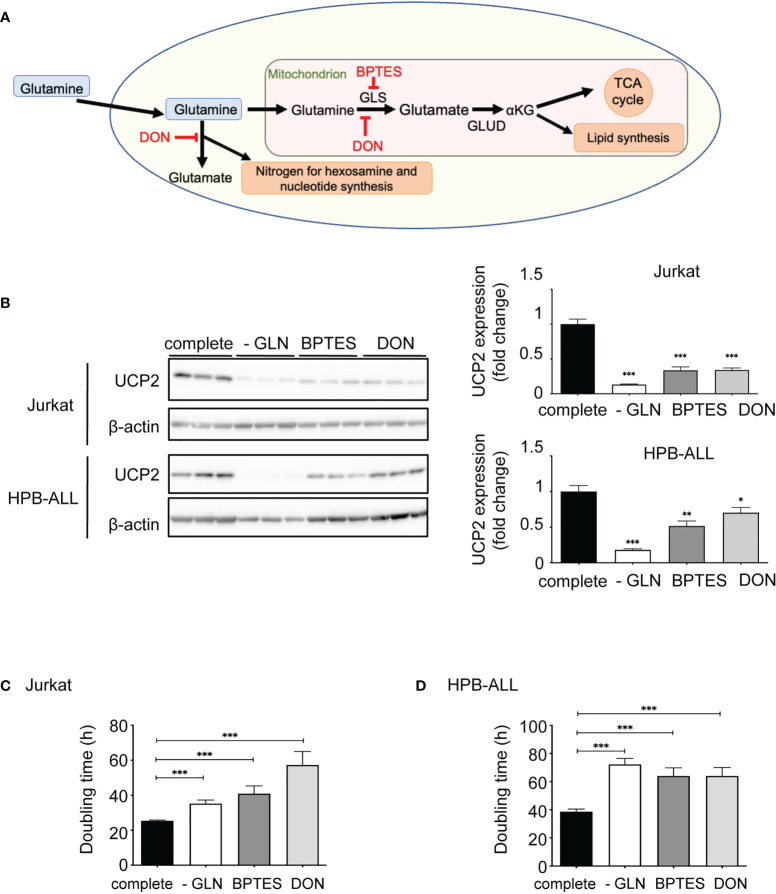
Inhibition of glutamine metabolism in T-ALL cells decreases UCP2 steady-state expression. **(A)** Schematic representation of glutamine metabolism indicating targets of BPTES and DON. **(B)** Immunoblotting of UCP2 and β-actin in whole cell extracts of Jurkat and HPB-ALL cell lines after 72 hours of incubation in complete medium (complete), glutamine-deprived medium (-GLN), or in complete medium + BPTES (10 µM) or DON (0.05 µg/mL); quantification of UCP2 expression normalized to β-actin; data are expressed as mean ± SEM (n = 3). Doubling time in hours of **(C)** Jurkat and **(D)** HPB-ALL cells evaluated during 72 hours of culture in complete medium (complete), in glutamine-deprived medium (-GLN), or in complete medium + BPTES (10 µM) or DON (0.05 µg/mL); data expressed as mean ± SEM (n = 3). Statistical significance is represented as follows: * = vs. complete medium; ***p<0.001; **p<0.01; *p<0.05.

To further decipher the role of glutamine between direct availability and diverse metabolic pathways, we analyzed the expression of UCP2 after addition to the medium of two different inhibitors of glutamine metabolism: Bis-2-(5-phenylacetamido-1,3,4-thiadiazol-2-yl)ethyl sulfide (BPTES) and 6-Diazo-5-oxo-L-norleucine (DON) (**Figure 3A**). BTPES acts as a glutaminase (GLS) inhibitor, blocking the synthesis of glutamate from glutamine, while DON is a structural analogue of glutamine, which thus competes with glutamine at the level of several enzymes. Both inhibitors significantly decreased the expression of UCP2 in Jurkat and HPB-ALL cells (**Figure 3B**). In both cell lines, these drugs dramatically reduced proliferation rate, as effectively as glutamine deprivation (**Figures 3C, D**).

Pool of extracellular glutamine can also be modulated by L-Asparaginase (L-ASNase) (**Supplementary Figure 4A**), which is used in combination to other chemotherapies against acute lymphoblastic leukemia (ALL). This extracellular glutaminase activity of L-ASNase was recently shown to be crucial for its anti-leukemic function independently of the levels of expression of asparagine synthetase (ASNS) (35). In Jurkat cells, no change was observed in UCP2 levels following L-ASNase treatment (**Supplementary Figure 4B**). In contrast, treatment induced a decrease in UCP2 expression (**Supplementary Figure 4C**). Moreover, we observe a dramatic drop of cell proliferation in HPB-ALL cells compare to Jurkat cells (**Supplementary Figures 4E–D**). Our results show that impairment of proliferation following L-ASNase treatment in HPB-ALL correlated with loss of UCP2.

Altogether, our results establish that not only extracellular glutamine levels as well as cellular glutamine metabolism has a direct impact on UCP2 expression.

### UCP2 knockdown decreases cell proliferation and has a selective impact on respiration in T-ALL cell lines

**Figure 4:**
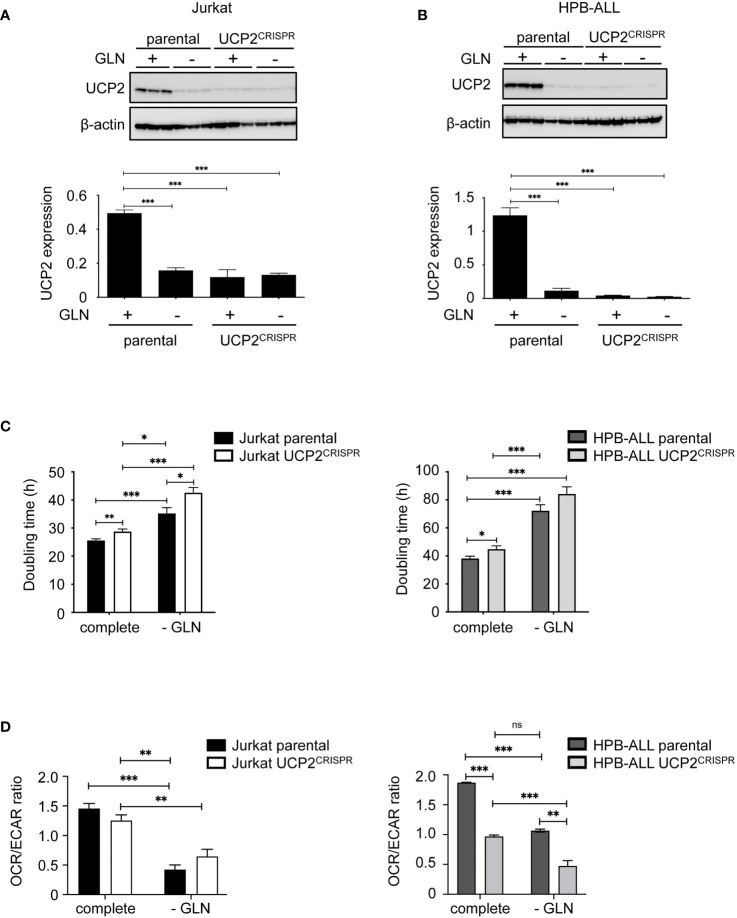
UCP2 knockdown decreases cell proliferation and has a selective impact on respiration in T-ALL cell lines. Immunoblot of UCP2 in whole cell extracts from either parental (n = 3) or UCP2^CRISPR^ (n = 3) Jurkat **(A)** and HPB-ALL **(B)** after 72 hours of incubation in complete medium (+) and glutamine-deprived medium (–); quantification of UCP2 expression normalized to β-actin and expressed as a mean ± SEM; significance: *** = p<0.001 * = vs. complete medium (+) parental cells. **(C)** Doubling time in hours as determined during 72 hours of culture in complete or glutamine-deprived medium (-GLN) for either parental or UCP2^CRISPR^ Jurkat and HPB-ALL; black bars = parental Jurkat cells; white bars = UCP2^CRISPR^ Jurkat cells; dark gray bars = parental HPB-ALL cells; light gray bars = UCP2^CRISPR^ HPB-ALL cells; data are expressed as mean ± SEM (n = 4). **(D)** The basal energy metabolism was assessed by analyzing the OCR/ECAR ratio measurement with SeaHorse XF96 equipment respectively in complete medium (complete) and in glutamine-deprived medium (-GLN), for either parental or UCP2^CRISPR^ Jurkat and HPB-ALL; black bars = parental Jurkat cells; white bars = UCP2^CRISPR^ Jurkat cells; dark gray bars = parental HPB-ALL cells; light gray bars = UCP2^CRISPR^ HPB-ALL cells; data are expressed as mean ± SEM (n = 4). Statistical significance is represented as follows: ***p<0.001; **p<0.01; *p<0.05. ns, not significant.

To further study the role of UCP2 expression, apart from glutamine deprivation, on cell proliferation and oxidative capacities, we generated CRISPR/cas9 UCP2 cell lines for Jurkat and HPB-ALL cells (UCP2^CRISPR^ Jurkat and UCP2^CRISPR^ HPB-ALL). As expected in complete medium, UCP2 expression was significantly decreased in UCP2^CRISPR^ Jurkat (**Figure 4A**) and UCP2^CRISPR^ HPB-ALL (**Figure 4B**) cells, by 76% and 96% of its respective level in parental cells. It should be noted that the residual level of UCP2 protein in the two types of UCP2^CRISPR^ cells is comparable to the one resulting from glutamine deprivation (**Figures 4A, B**). Then, cell proliferation and the ratio between mitochondrial respiration and glycolysis (OCR/ECAR) were investigated in these UCP2-silenced cells. First, in complete medium, UCP2 silencing significantly decreased cell proliferation and enhanced inhibition of cell proliferation triggered by glutamine deprivation in Jurkat and HPB-ALL cells (**Figure 4C**). Of note, in HPB-ALL cells, the increase in doubling time triggered by the silencing of UCP2 in the absence of glutamine did not reach significance, which could be explained by the already high doubling time of the HPB-ALL control cells in this culture condition (parental 72.22 ± 4.333 hours, n=9, vs. UCP2^CRISPR^ 84.17 ± 5.121 hours, n=12; P = 0.1051). Interestingly, while no significant change in the OCR/ECAR ratio was observed in Jurkat cells after UCP2 silencing whatever the culture conditions, a massive decrease in this metabolic ratio was observed in UCP2^CRISPR^ HPB-ALL cells (**Figure 4D**). In fact, UCP2 silencing in HPB-ALL cells significantly reduced both their basal and maximal respiration in complete medium or after glutamine deprivation (**Supplementary Figures 5A, 5B**). In contrast, Jurkat cells, relying massively in glycolysis, have no respiration alteration after UCP2 silencing (**Supplementary Figures 5A, 5B**).

Thus, while UCP2 silencing decreases cell proliferation in all leukemia cells, it also alters mitochondrial respiration of T-ALL cells relying on oxidative metabolism dependent of glutamine.

### UCP2 knockdown rewires HPB-ALL metabolism towards glycolysis

**Figure 5:**
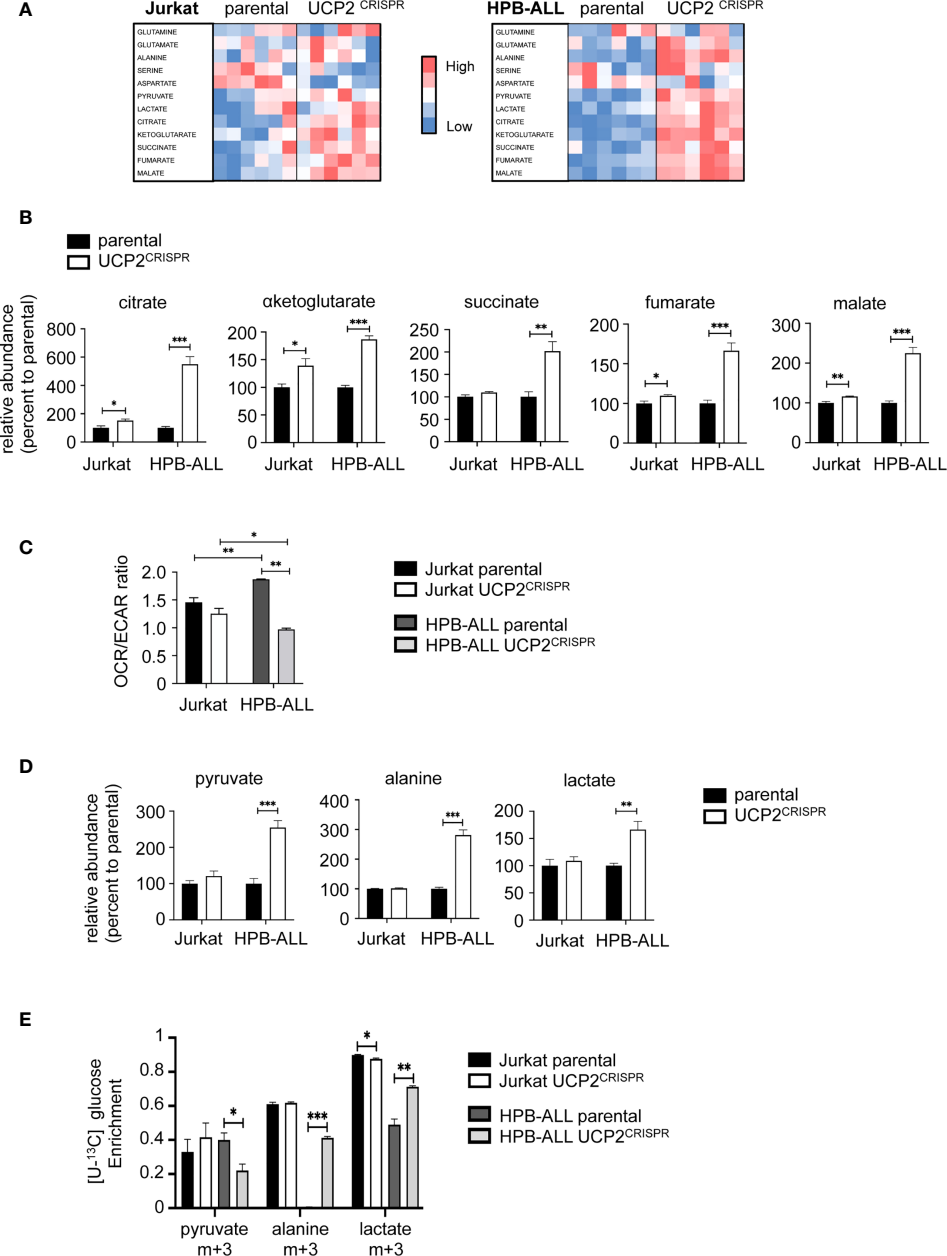
UCP2 knockdown rewires HPB-ALL metabolism towards glycolysis. **(A)** Jurkat cells and HPB-ALL cells and their corresponding UCP2^CRISPR^ cells were cultured in complete medium. Relative abundance of 12 metabolites measured by liquid chromatography–tandem mass spectrometry (LC-HRMS) is visualized as heat map representation (blue color corresponds to low values and red to high values) by distinguishing the number of samples for each condition. **(B)** The relative abundance of TCA cycle metabolites measured by liquid chromatography-tandem mass spectrometry (LC-HRMS) is represented as a percentage relative to parental. Relative abundance of citrate, αketoglutarate, succinate, fumarate and malate were determined in Jurkat cells and HPB-ALL cells (n = 6) (black bars); UCP2^CRISPR^ Jurkat (n = 6) and UCP2^CRISPR^ HPB-ALL cells (n = 4) (white bars). **(C)** The basal energy metabolism was assessed by analyzing the OCR/ECAR ratio measurement with SeaHorse XF96 equipment respectively in complete medium for either parental or UCP2^CRISPR^ Jurkat and HPB-ALL cells; black bars = parental Jurkat cells; white bars = UCP2^CRISPR^ Jurkat cells; dark gray bars = parental HPB-ALL cells; light gray bars = UCP2^CRISPR^ HPB-ALL cells; data are expressed as mean ± SEM (parental n = 3 and UCP2^CRISPR^ n = 6). **(D)** The relative abundance of pyruvate, alanine and lactate measured by LC-HRMS was represented as a percentage relative to parental cells, in Jurkat and HPB-ALL cells (n = 6) (black bars); UCP2^CRISPR^ Jurkat cells (n = 6) and UCP2^CRISPR^ HPB-ALL cells (n = 4) (white bars). Data are expressed as mean ± SEM. **(E)** The enrichment of (m+3) pyruvate, (m+3) alanine and (m+3) lactate indicates the part derived from the oxidation of [U-^13^C] glucose for either parental or UCP2^CRISPR^ Jurkat and HPB-ALL cells; black bars = parental Jurkat cells; white bars = UCP2^CRISPR^ Jurkat cells; dark gray bars = parental HPB-ALL cells; light gray bars = UCP2^CRISPR^ HPB-ALL cells; data are expressed as mean ± SEM (n = 3). Statistical significance is represented as follows: ***p<0.001; **p<0.01; *p<0.05.

To understand whether and how UCP2 differentially impacted metabolic adaptations depending on T-ALL cell types, we measured by LC-HRMS the levels of various metabolites belonging to glycolysis and mitochondrial metabolism in parental and UCP2^CRISPR^ cells. Heat map representations of the relative metabolite abundance revealed that UCP2 silencing had a higher impact in HPB-ALL cells than in Jurkat cells (**Figure 5A**). In particular, the levels of key metabolites of the TCA cycle such as citrate, αketoglutarate, succinate, fumarate and malate were strongly enhanced in HPB-ALL cells upon UCP2 silencing while they were slightly altered in Jurkat cells (**Figure 5B**). These results were reinforced by the global metabolic rewiring shown by the OCR/ECAR ratio observed in HPB-ALL cells but not in Jurkat cells (**Figure 5C**). As pyruvate, lactate and alanine are an easy read-out of glycolysis, we closely examined their relative abundance in each cell line. In Jurkat cells, UCP2 silencing did not alter pyruvate, alanine and lactate levels whereas these metabolites were significantly increased in HPB-ALL cells (**Figure 5D**). In addition, in UCP2^CRISPR^ HPB-ALL cells, enrichments of (m+3) alanine and (m+3) lactate from [U-^13^C] glucose were significantly increased (**Figure 5E**), while those for (m+2) fumarate, (m+2) malate and (m+2) aspartate were slightly decreased (**Supplementary Figure 6A**). In agreement with this metabolic rewiring, analysis of Nuclear Magnetic Resonance (NMR) spectroscopy using Orthogonal Projections to Latent Structures Discriminant Analysis (OPLS-DA) clearly revealed a distinct chemical composition of the extracellular medium and intracellular content between the parental and the UCP2^CRISPR^ HPB-ALL (**Supplementary Figures 6B, C**). Consistent with our previous results, an increase in extracellular and intracellular lactate was observed in UCP2^CRISPR^ HPB-ALL cells compared to that of parental cells reflecting an increase of glycolysis induced by UCP2 silencing (**Supplementary Figures 6D, E**).

Altogether, our results show that glutaminergic HPB-ALL cells reprogram their metabolism upon metabolic challenges in a UCP2-dependent manner, in contrast to glycolytic Jurkat cells, which adapt their metabolism independently of UCP2 expression. These results highlight a specific function of UCP2 upon metabolic stress based on the intrinsic metabolic plasticity of the T-ALL cells.

### DM-αKetoglutarate but not DM-Malate rescues metabolic rewiring following UCP2 knockdown

**Figure 6:**
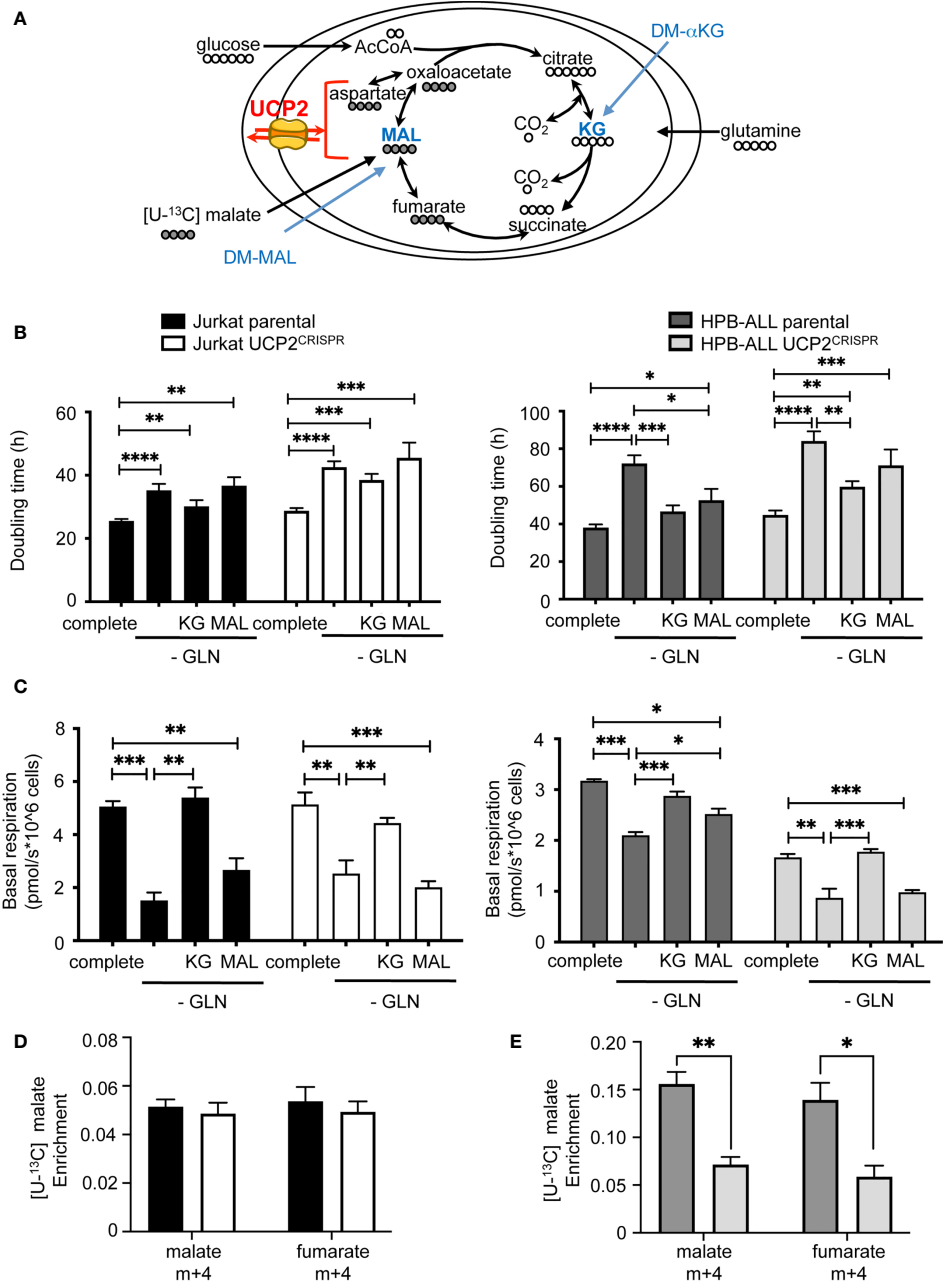
DM-αKetoglutarate but not DM-Malate rescues metabolic rewiring following UCP2 knockdown. **(A)** Schematic representation of the TCA cycle with entries for cell-permeant DM-αKetoglutarate (KG) and DM-Malate (MAL); the number of carbons of the different intermediates is indicated; UCP2 transports four carbon intermediates. **(B)** Doubling time rate in hours; black bars = parental Jurkat cells; white bars = UCP2^CRISPR^ Jurkat cells; dark gray bars = parental HPB-ALL cells; light gray bars = UCP2^CRISPR^ HPB-ALL cells; complete medium (complete) and glutamine-deprived medium (-GLN) data are shown; KG = 2 mM DM-αKG added to the medium; MAL = 2 mM DM-MAL added to the medium; data are expressed as mean ± SEM (n = 3 - 12). **(C)** Basal mitochondrial respiration rate measurement with SeaHorse XF96 equipment expressed in picomoles per second and millions of cells for either parental or UCP2^CRISPR^ Jurkat and HPB-ALL cell lines; black bars = parental Jurkat cells; white bars = UCP2^CRISPR^ Jurkat cells; dark gray bars = parental HPB-ALL cells; light gray bars = UCP2^CRISPR^ HPB-ALL cells; complete medium (complete) and glutamine-deprived medium (-GLN) data are shown; KG = 2 mM DM-αKG added to the medium; MAL = 2 mM DM-MAL added to the medium; data are expressed as mean ± SEM (n = 3 - 6). Enrichment of (m+4) malate and (m+4) fumarate indicates the portion derived from [4-^13^C] malate metabolism for either parental or UCP2^CRISPR^ Jurkat **(D)** and HPB-ALL **(E)** cell lines; black bars = parental Jurkat cells; white bars = UCP2^CRISPR^ Jurkat cells; dark gray bars = parental HPB-ALL cells; light gray bars = UCP2^CRISPR^ HPB-ALL cells; data are expressed as mean ± SEM (n = 3). Statistical significance is represented as follows: ***p<0.001; **p<0.01; *p<0.05; ****p<0.0001.

As glutamine-dependent T-ALL is affected by UCP2 in cellular metabolic adaptation, we investigated whether supplementing cells with αketoglutarate or malate would prevent the UCP2-dependent metabolic adaptation observed after glutamine deprivation. Although these metabolites require a carrier to be exported out or imported into the mitochondria, dimethyl-αketoglutarate (DM-αKG) and dimethyl-malate (DM-Mal) are cell permeable analogs (**Figure 6A**). Measurements of the doubling time under all these conditions revealed a different proliferative response to KG or MAL supply between the two cell types. Indeed, in both parental and UCP2^CRISPR^ Jurkat cells, neither addition of KG nor MAL rescued the decrease in proliferation triggered by glutamine deprivation (**Figure 6B**). In contrast, in parental HPB-ALL cells, both KG and MAL rescued the inhibition of cell proliferation (**Figure 6B**). However, in UCP2^CRISPR^ HPB-ALL cells, KG only partially rescued cell proliferation while MAL had no significant effect (**Figure 6B**). Interestingly, different effects of KG and MAL were observed on mitochondrial respiration. Indeed, in Jurkat cells, KG but not MAL was able to rescue basal and maximal mitochondrial respiration decreased following glutamine deprivation. As observed for cell proliferation, UCP2 silencing had no effect on these metabolic variations (**Figure 6C** and **Supplementary Figure 7A**). In HPB-ALL cells while both KG and MAL are able to rescue respiration, this rescue by MAL is abolished when silencing of UCP2 (**Figure 6C** and **Supplementary Figure 7B**). To decipher how UCP2 expression modulates malate metabolism, we realized fluxomic experiments using [U-^13^C] malate and analyzed its intracellular abundance and its conversion to fumarate. First, HPB-ALL cells displayed three times higher level of malate than Jurkat cells in the presence of UCP2. Interestingly, in HPB-ALL cells but not in Jurkat cells, malate level was significantly reduced after UCP2 silencing (**Figures 6D, E**). In both cell lines, all malate was converted to fumarate (**Figure 6D**). However, the conversion of malate in fumarate was not altered by UCP2 silencing in Jurkat cells while it was significantly affected in UCP2^CRISPR^ HPB-ALL cells. Strikingly, in these cells and in the absence of UCP2, both the abundance of malate and its conversion to fumarate reached those of Jurkat cells whose metabolism rely highly in glycolysis, and it is independent of UCP2.

Altogether, our results demonstrate that the proliferative effect of KG is distinct from its effect on mitochondrial respiration depending on the dependency of the cell type on glutamine for respiration. Indeed, while the increase of respiration is sufficient to compensate the loss of proliferation in HPB-ALL, this was not the case in Jurkat cells. Moreover, in HPB-ALL mitochondrial respiration, replenishment by KG in UCP2^CRISPR^ cells is not sufficient to recover parental respiration.

These results highlight the essential role of UCP2 in glutamine-dependent mitochondrial respiration to support proliferation in T-ALL.

## Discussion

In this study, we reveal the importance of evaluating energetic metabolism of leukemia cells to be able to efficiently target their proliferation. T-ALL cells can rely on different metabolisms, such as oxidative metabolism for HPB-ALL cells and glycolysis for Jurkat cells. We report that UCP2 plays a key role in controlling leukemia cell proliferation through glutamine metabolic remodeling. In HPB-ALL cells, UCP2 knockdown decreased oxygen consumption but more importantly rewired cellular metabolism to glycolysis such as glutamine deprivation. Furthermore, we demonstrate, through mitochondrial *in vitro* replenishment by DM-αKG and DM-Mal, and [U-^13^C] malate fluxes, that UCP2 promotes TCA cycle function and glutamine bioenergetics in HPB-ALL cells through its role as a malate transporter. In Jurkat cells which express low levels of UCP2 and strongly rely on glycolysis compared to HPB-ALL, UCP2 silencing does not alter respiration but impacts proliferation.

T-ALL cells are commonly known to primarily rely on glutamine availability for survival and proliferation (26). Glutamine contributes to many essential processes in proliferating tumor cells. Indeed, it participates in the *de novo* biosynthesis of both purines and pyrimidines, and in fueling the TCA cycle as a primary carbon and energy source. It also supports cellular defenses against oxidative stress and supplements glucose metabolism in the production of macromolecules (36). Several oncogenes, including Notch (26, 37), c-Myc (38) or PTEN (39, 40), control metabolism in order to support cell proliferation. Metabolic treatments for leukemia typically target glutaminolysis (41–44). However, we presently show that altering glutamine metabolism will force T-ALL cells to use glycolysis to survive. This underlines the plasticity of T-ALL cells which allows putative relapse following treatment. Thus, relapses and clonal selection of resistant cells remain recurrent issues in leukemias such as T-ALL and AML (1).

In this context, we evaluated whether UCP2 protein could be a good marker to determine metabolism of T-ALLs and a good target for treatment. An important source of confusion regarding the evaluation of UCP2, particularly in respect to transcriptomic data from tumor cells, is its peculiar translational regulation (29) due to constant repression (27). In fact, we previously reported that glutamine availability directly increased UCP2 translation (27). In our study, we investigated how UCP2 controls T-ALL cell bioenergetics and proliferation using two complementary approaches, glutamine deprivation and UCP2 knock-down using the CRISPR technology. Interestingly, with glutamine deprivation, we demonstrate for the first time that glutamine metabolism (i.e. steps down to glutaminase) is as important as glutamine availability (**Figure 3B**) for the regulation of UCP2 protein in T-ALL cells. In the absence of glutamine, addition of permeable forms of Krebs cycle intermediates to the external medium differently restores the respiration rates of parental cells. Moreover, while both DM-αKG and DM-Malate fuel the respiratory chain, DM-Malate was only effective in HPB-ALL cells, highlighting their dependency on mitochondrial bioenergetics. We show that Jurkat cells have a more flexible metabolism and are able to overcome better, at metabolic level, loss of UCP2 compared to HPB-ALL cells. Indeed, Jurkat cells have the capacity to adapt their metabolism to redirect glucose to metabolic pathways that require substrate. Therefore, there is enough compensation to not appreciate significant changes in respiration. However, importantly, we show that UCP2 knockdown alters the proliferation of both T-ALL cell lines. In UCP2 knockdown HPB-ALL cells, the oxidation of glutamine is not efficient leading to a decrease in proliferation associated with decreased respiration and metabolic reprogramming promoting glycolysis. Whereas Jurkat cells are less dependent on glutamine bioenergetics to proliferate, UCP2 knockdown, by affecting TCA cycle fueling, may alter glutamine metabolism for biosynthetic precursors of purines and pyrimidines and fatty acid synthesis through reductive carboxylation. Moreover, we also show that UCP2 knockdown-dependent metabolic reprogramming functions independently of Notch1 expression and its role in promoting glutamine metabolism. Our results emphasize that metabolic alteration and/or substrates addiction outweighs oncogenic hits.

**Figure 7:**
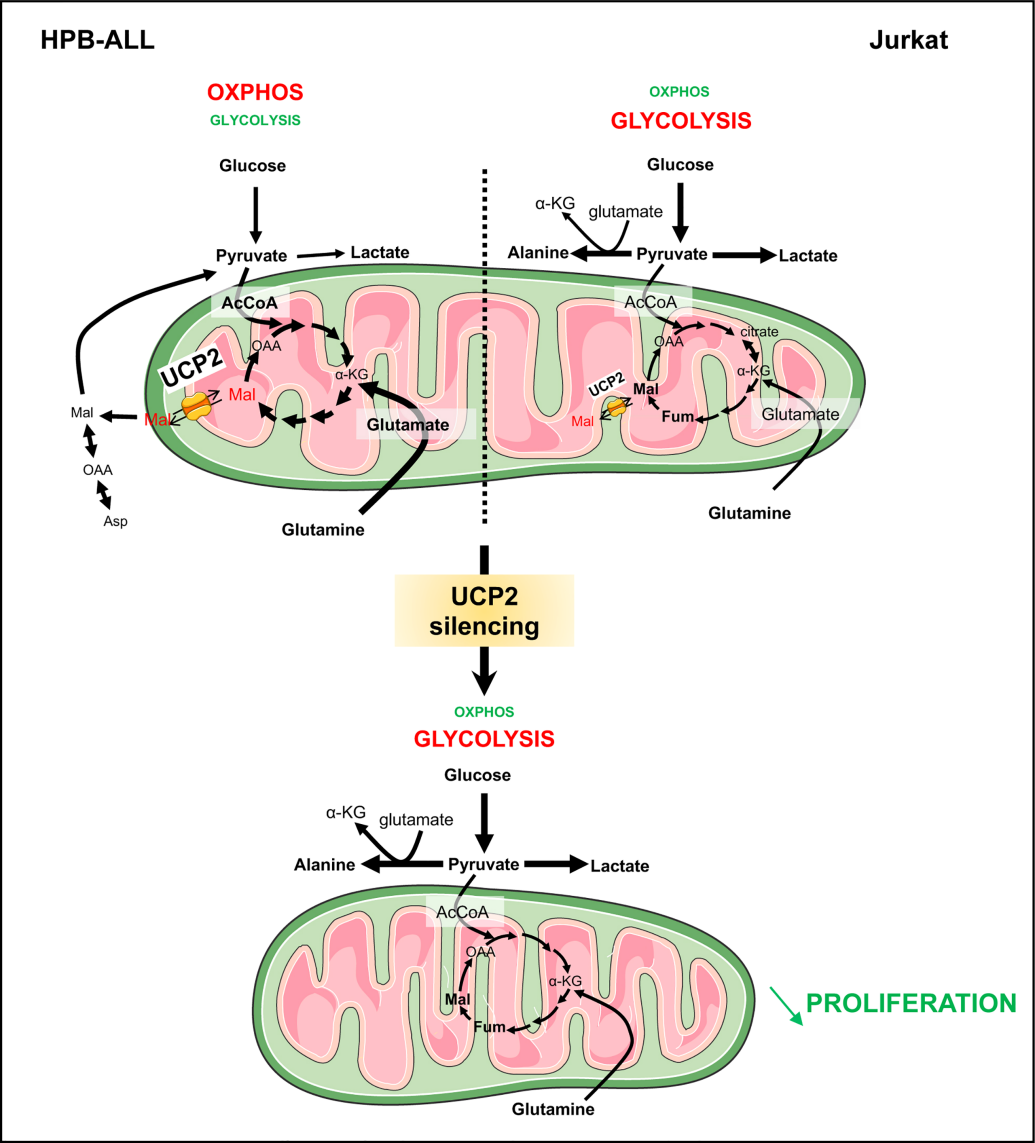
UCP2 allows proper use of glutamine to fulfill the TCA cycle in T-ALLs. Schematic view of UCP2 function in leukemia cells relying more in oxidative metabolism (HPB-ALL) and in glycolysis (Jurkat). HPB-ALL cells consume two glutamine molecules; the two molecules enter the TCA cycle and transform into malate; thereafter, one malate remains in the TCA cycle leading to synthesis of oxaloacetate; the other one is exported by UCP2 into the cytosol where it is transformed into oxaloacetate then into aspartate and also into pyruvate *via* the malic enzyme. UCP2 allows efficient use of glutamine to fulfill the TCA cycle and, at the same time, maintain leukemia cell proliferation. Instead, Jurkat cells consume glutamine to generate citrate and glucose to fulfill glycolysis. Following UCP2 silencing, the export of malate is reduced, which induces blockade of the TCA cycle (i.e. accumulation of fumarate and malate) that will force the HPB-ALL cell to reorient its metabolism towards glycolysis. The rewiring of metabolism induced by UCP2 loss is associated with a decreased proliferation of leukemia cells.

In UCP2 knockdown HPB-ALL cells, DM-αKG supplementation remains effective while DM-MAL is no longer able to rescue respiration in the absence of glutamine. Of note DM-MAL is unable to restore maximal respiration in absence of glutamine, suggesting that DM-MAL supplementation acts directly as substrate supply and is not sufficient to modify mitochondria content and per se mitochondrial reserve capacity unlike DM-αKG. However, DM-αKG supplementation is not efficient in restoring respiration levels in UCP2^CRISPR^ HPB-ALL cells comparable to those in parental cells. This result could be explained by an obstruction of the TCA in the UCP2 knockdown condition, caused by the accumulation of TCA intermediates such as succinate, fumarate, malate (**Figure 5B**) and is associated with a decrease in proliferation. In UCP2 knockdown cells, the oxidation of glutamine is not efficient leading to a decrease in proliferation associated with metabolic reprogramming promoting glycolysis (**Figure 7**). Altogether, our results further strengthen our previous studies linking UCP2 and energy metabolism (17, 18). In addition, our results further confirm the role of UCP2 within the frame of the TCA cycle as a metabolite transporter like all other members of the mitochondrial carrier family (25). Indeed, in liposome exchange experiments, Vozza et al. demonstrated that malate is efficiently exported by UCP2 in exchange for phosphate plus a proton (25). Recently, the same team demonstrated in pancreatic cancer cells that UCP2 catalyzes the efflux of mitochondrial aspartate. In agreement with our work on colorectal cancer (20), they showed that the role of UCP2 in ROS handling is related to its substrate transport function rather than uncoupling activity (24).

Major issues in leukemia treatment remain in clonal selection of resistant cells allowing relapses. In AML, resistance to cytarabine chemotherapy has been associated to increased oxidative phosphorylation and the use of inhibitors of the electron transport chain *in vitro* and *in vivo* sensitized cells to chemotherapy treatment (45). In line with this observation, determining the metabolism of T-ALL could allow us to decipher synergistic treatments. Interestingly, inhibition of mitochondrial complex I with metformin, leading to decreased oxidative capacity, has been reported to synergize with daunorubicin, vincristine, L-asparaginase, and etoposide, enhancing the antileukemic effects of these drugs (46–48).

In conclusion, our study demonstrates that UCP2 plays an important role in leukemia cells to allow proper use of glutamine, in particular when the cells have a high energetic dependency on glutaminolysis. Further experiments using different leukemic cell lines and human leukemia samples would be interesting to demonstrate whether the expression of the mitochondrial protein UCP2 could be a good marker to stratify leukemia cells whose oxidative metabolism depends on glutamine. Thus, metabolic targeting through inhibition of UCP2 could be considered as an interesting strategy to synergize antileukemic treatment.

## Data availability statement

The original contributions presented in the study are included in the article/**Supplementary Material**. Further inquiries can be directed to the corresponding author.

## Author contributions

TS and M-CA-G. conceived the project, designed and performed experiments and interpreted results. GB, RD, CP-B, and CPe also aided with experimental design and interpretation of results. OR, AL, CC, VL, CR, and M.C. performed and analyzed experiments. RD provided administrative, technical and material support. The manuscript was written by M-CA-G and edited by TS, EA, CPo, RD, CP-B, and CPe. The study was supervised by M-CA-G. All authors contributed to the article and approved the submitted version.

## Funding

TS was supported by the doctoral fellowship from Paris Diderot and by the European Research Council (ERC-2013-StG-336629). This work was financially supported by grants from “La Ligue contre le Cancer” (LCC RS17/75-63), the “Fondation ARC” (PJA 20151203347), the “Centre National de la Recherche Scientifique” (CNRS), the “Institut National de la Santé et de la Recherche Médicale” (Inserm) and the University Paris Cité.

## Acknowledgments

We gratefully thank Frédéric Bouillaud and Anne Lombès for helpful discussion and support of this project. We thank Pfumio’s laboratory for kindly given malignant human T-ALL cells. We thank Gisèle Froment, Didier Nègre and Caroline Costa from the lentivectors production facility/SFR BioSciences Gerland - Lyon Sud (UMS3444/US8). We thank the metabolic platform Mikaël Croyal and Audrey Aguesse from the Mass Spectrometry platform of Nantes University for expert technical assistance. We thank MetaboParis-Santé (convention N° EX023598), an action supported by the Île-de-France Region, Université de Paris, CNRS and Inserm, and Nicolas Giraud for the access to the NMR equipment.

## Conflict of interest

The authors declare that the research was conducted in the absence of any commercial or financial relationships that could be construed as a potential conflict of interest.

## Publisher’s note

All claims expressed in this article are solely those of the authors and do not necessarily represent those of their affiliated organizations, or those of the publisher, the editors and the reviewers. Any product that may be evaluated in this article, or claim that may be made by its manufacturer, is not guaranteed or endorsed by the publisher.

## Abbreviations

ASNS, asparagine synthetase; BPTES, Bis-2-(5-phenylacetamido-1,3,4-thiadiazol-2-yl)ethyl sulfide; CS, citrate synthase; DM-αKG, dimethyl-α ketoglutarate; DM-malate, dimethyl-malate; DON, 6-Diazo-5-oxo-L-norleucine; ECAR, extracellular acidification rate; GLS, glutaminase; L-ASNase, L-Asparaginase; LC-HRMS, Liquid Chromatography-High Resolution Mass Spectrometry; NMR, Nuclear Magnetic Resonance; OCR, oxygen consumption rate; PDL, poly D lysine; T-ALL, T-cell acute lymphoblastic leukemia; TCA, tricarboxylic acid cycle; UCP2, UnCoupling Protein 2.
