# Supplementary figures and images for: UCP2 silencing restrains leukemia cell proliferation through glutamine metabolic remodeling
Source: Front Immunol. 2022 Oct 6;13:960226. doi: 10.3389/fimmu.2022.960226 (PMC9582289; doi:10.3389/fimmu.2022.960226)

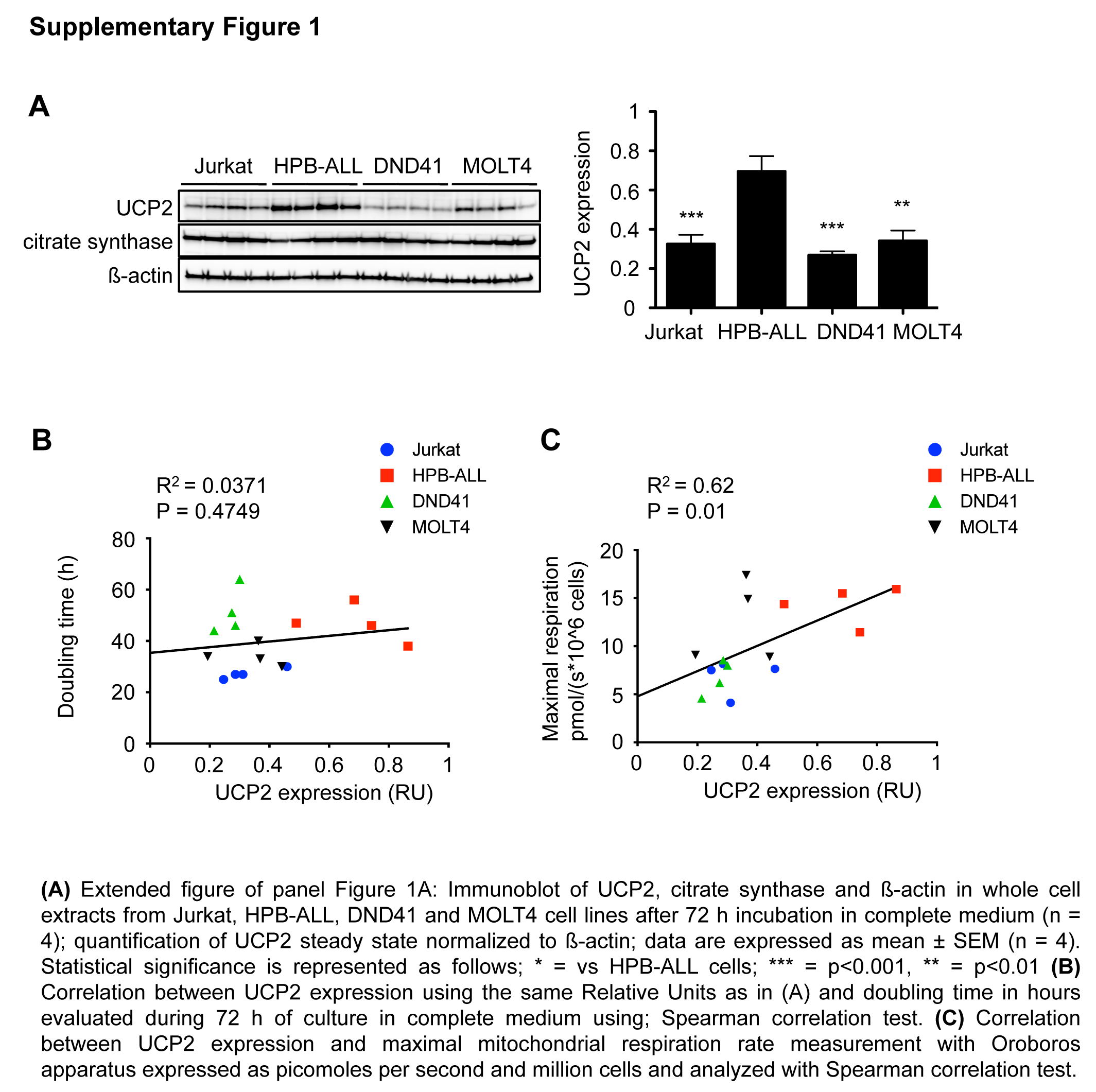

Supplement: Supplementary file 1 [file Image_1.jpeg]

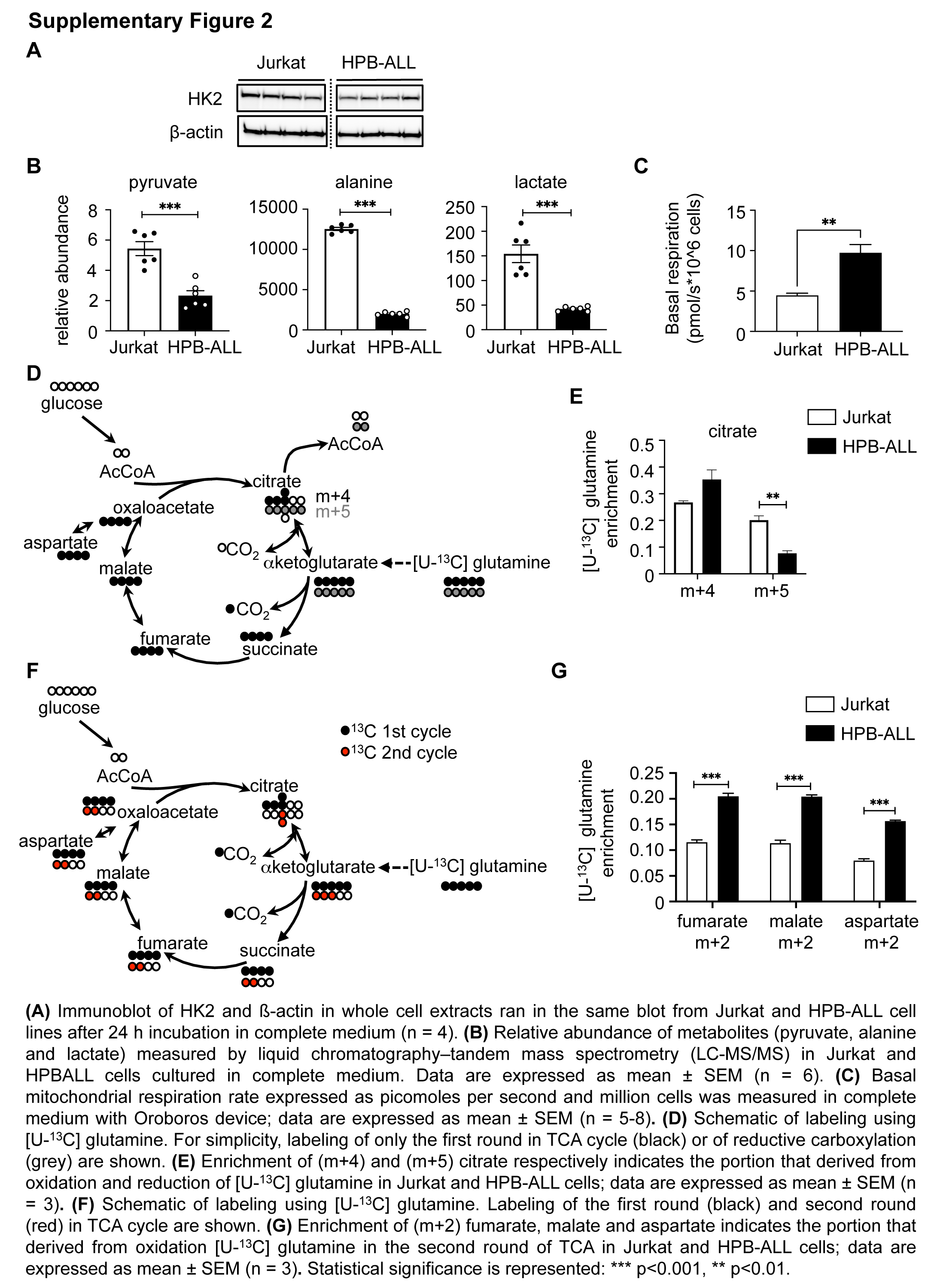

Supplement: Supplementary file 2 [file Image_2.jpeg]

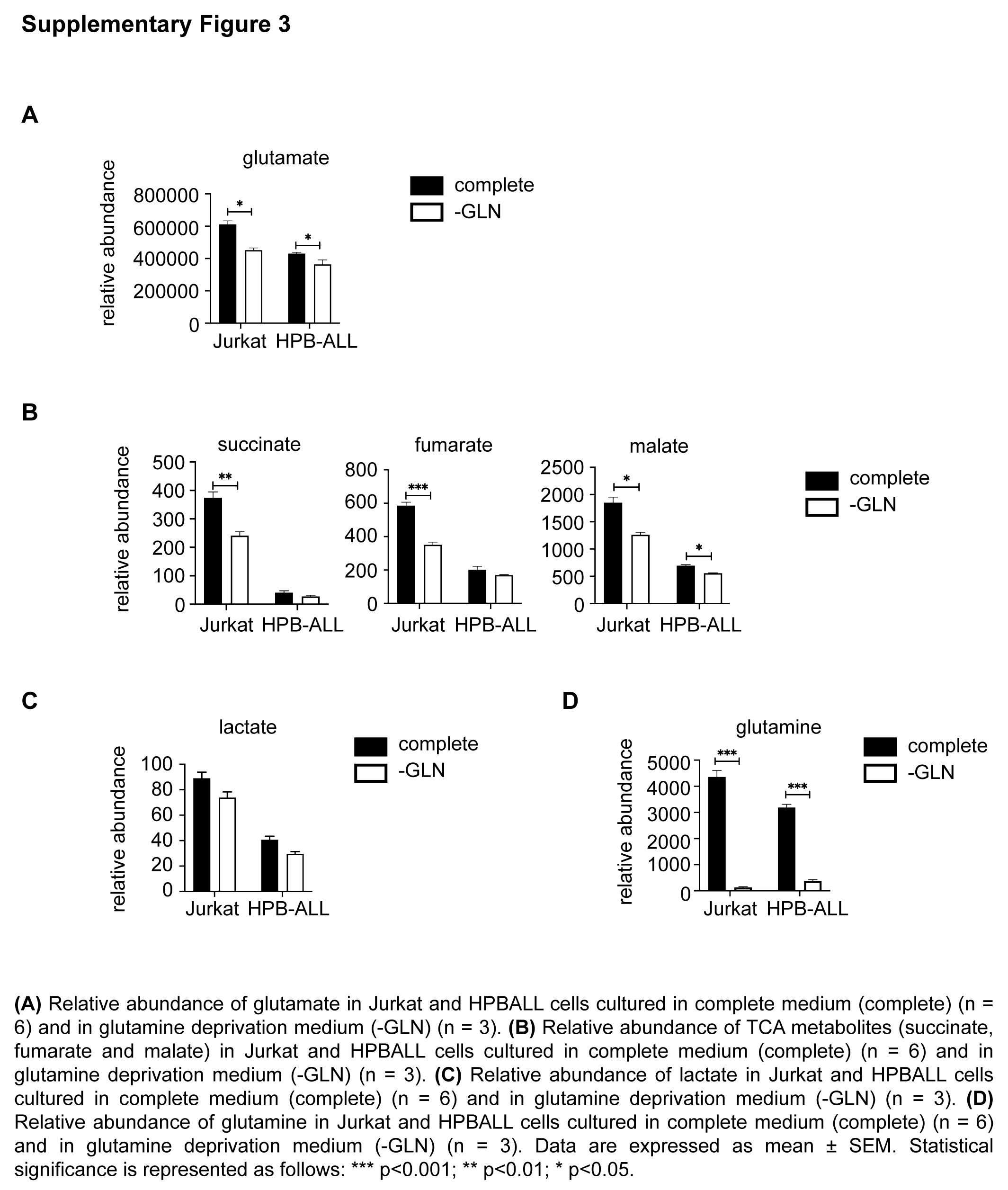

Supplement: Supplementary file 3 [file Image_3.jpeg]

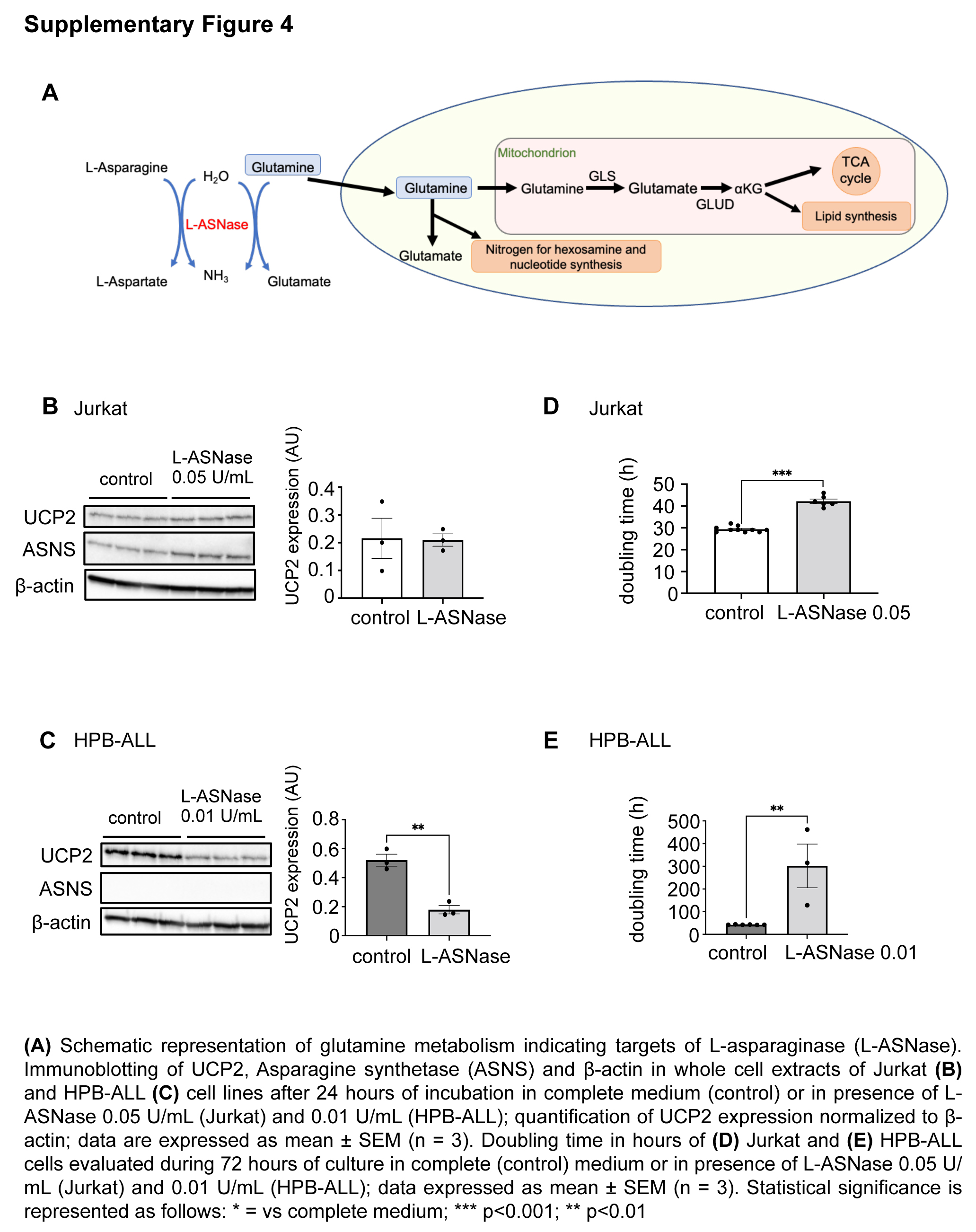

Supplement: Supplementary file 4 [file Image_4.jpeg]

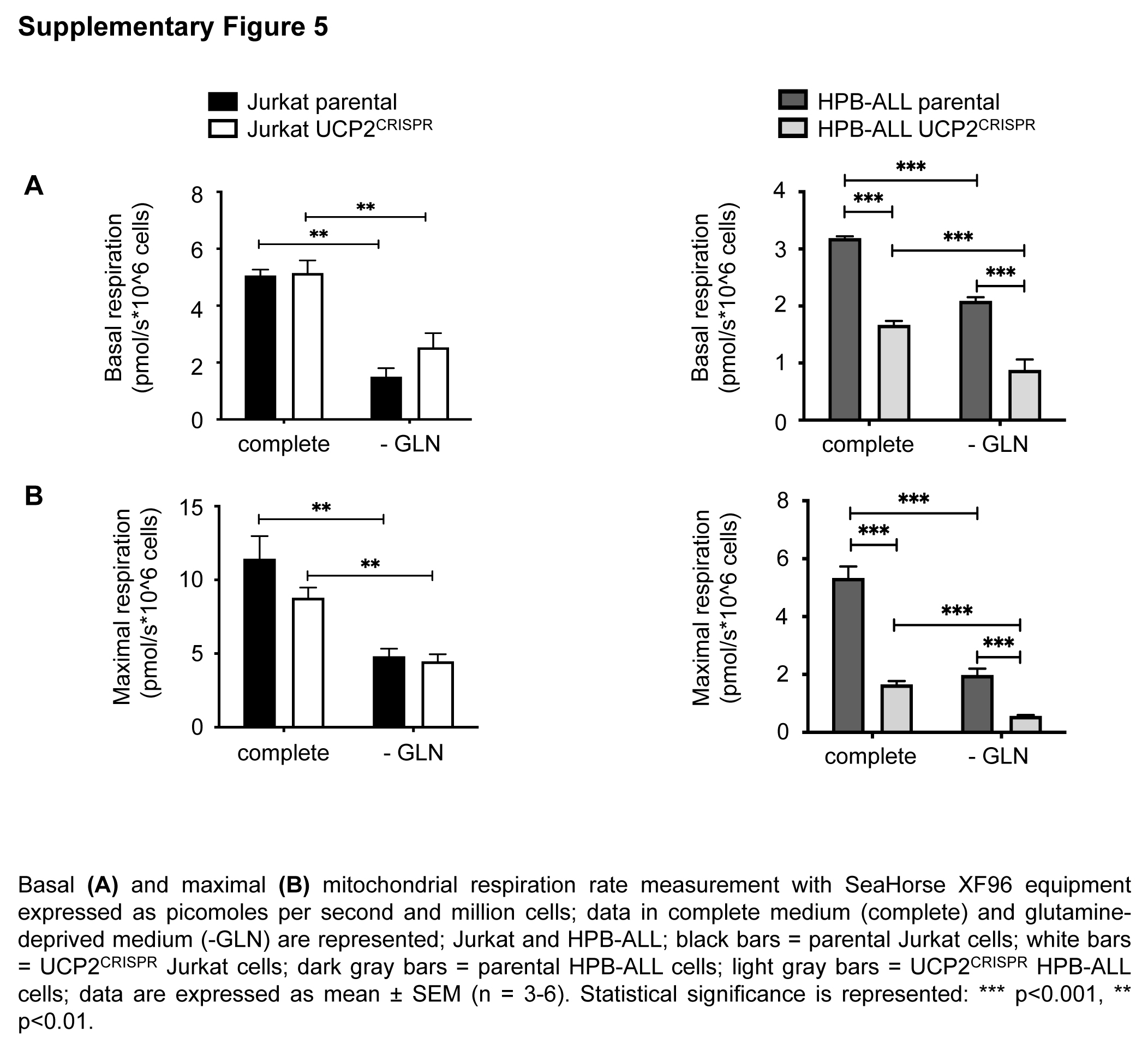

Supplement: Supplementary file 5 [file Image_5.jpeg]

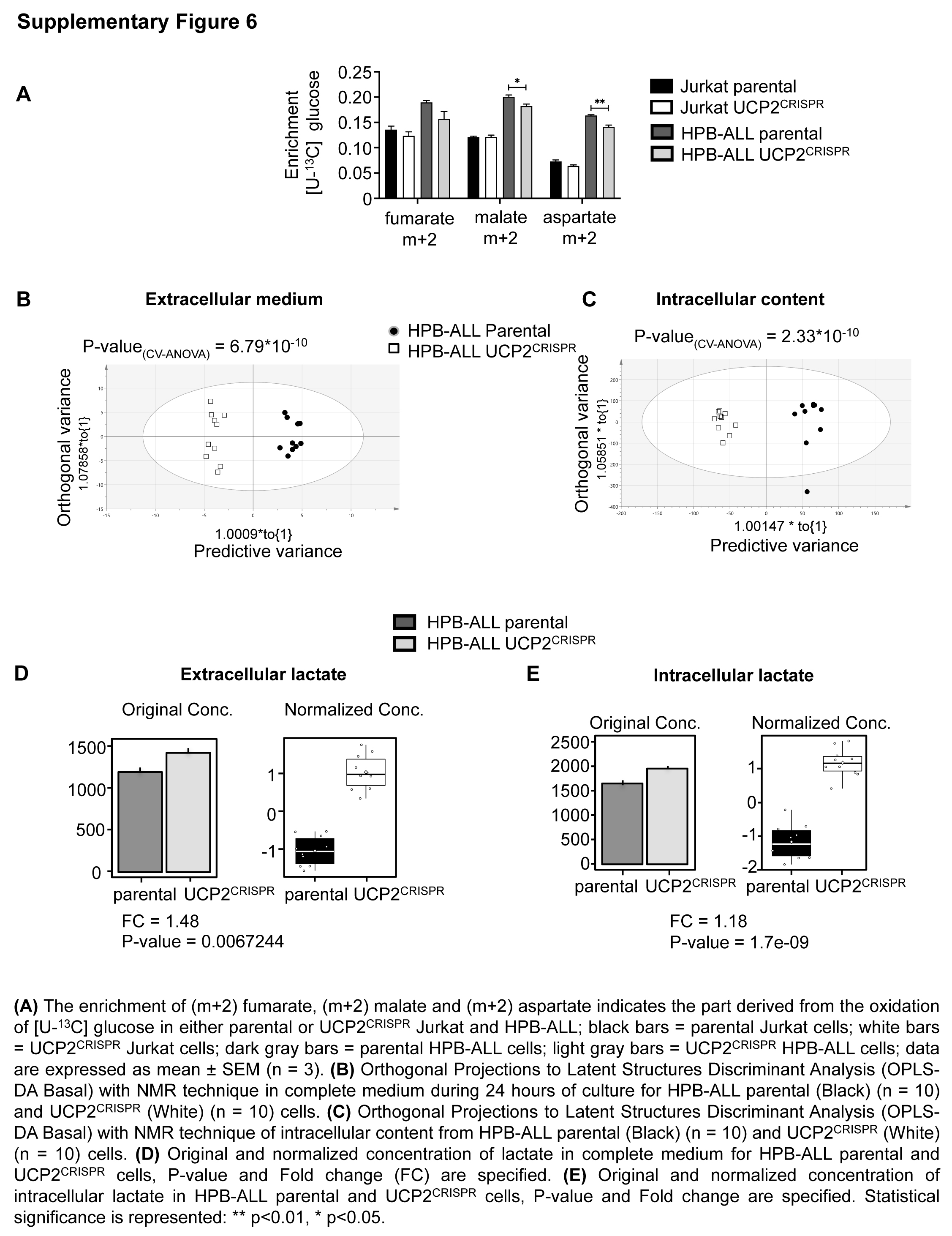

Supplement: Supplementary file 6 [file Image_6.jpeg]

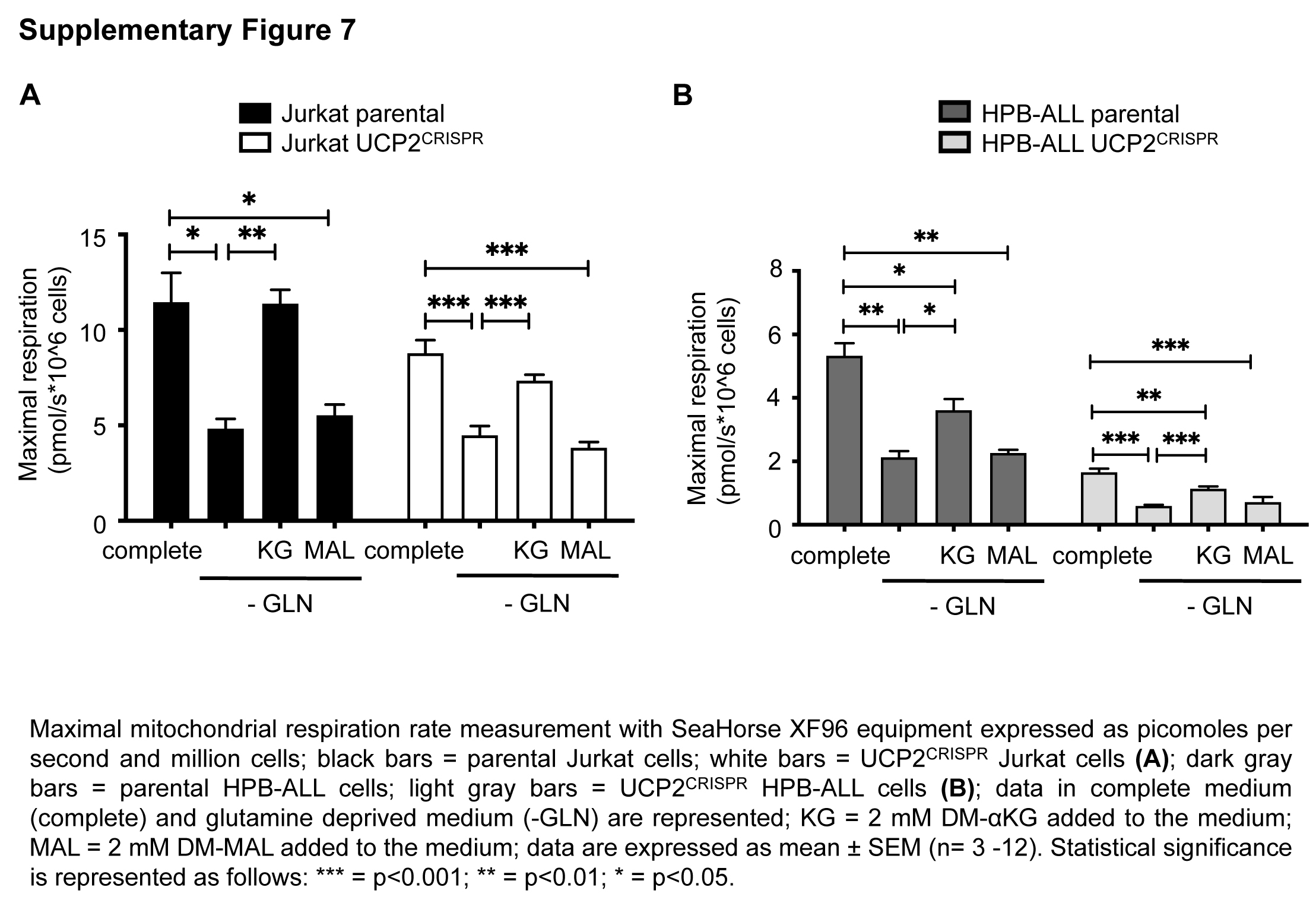

Supplement: Supplementary file 7 [file Image_7.jpeg]
